# Phenazopyridine-Induced Methemoglobinemia in a Jehovah’s Witness Treated with High-Dose Ascorbic Acid Due to Methylene Blue Contradictions: A Case Report and Review of the Literature

**DOI:** 10.3390/hematolrep15020034

**Published:** 2023-05-24

**Authors:** Sasmith R. Menakuru, Vijaypal S. Dhillon, Mona Atta, Keeret Mann, Ahmed Salih

**Affiliations:** Department of Internal Medicine, Indiana University School of Medicine-Muncie, Muncie, IN 47306, USA

**Keywords:** methemoglobinemia, ascorbic acid, phenazopyridine, methylene blue

## Abstract

Methemoglobinemia is an acute medical emergency that requires prompt correction. Physicians should have a high degree of suspicion of methemoglobinemia in cases that present with hypoxemia that does not resolve with supplemental oxygenation, and they should confirm this suspicion with a positive methemoglobin concentration on arterial blood gas. There are multiple medications that can induce methemoglobinemia, such as local anesthetics, antimalarials, and dapsone. Phenazopyridine is an azo dye used over-the-counter as a urinary analgesic for women with urinary tract infections, and it has also been implicated in causing methemoglobinemia. The preferred treatment of methemoglobinemia is methylene blue, but its use is contraindicated for patients with glucose-6-phosphatase deficiency or those who take serotonergic drugs. Alternative treatments include high-dose ascorbic acid, exchange transfusion therapy, and hyperbaric oxygenation. The authors report a case of a 39-year-old female who took phenazopyridine for 2 weeks to treat dysuria from a urinary tract infection and subsequently developed methemoglobinemia. The patient had contraindications for the use of methylene blue and was therefore treated with high-dose ascorbic acid. The authors hope that this interesting case promotes further research into the utilization of high-dose ascorbic acid for managing methemoglobinemia in patients who are unable to receive methylene blue.

## 1. Introduction

Methemoglobin (MetHb) is an oxidized form of hemoglobin (Hb), in which the heme iron configuration changes from the ferrous (Fe^2+^) to the ferric (Fe^3+^) state. Due to iron being in the ferric state, allosteric changes occur in methemoglobin, which permit oxygen to bind irreversibly. This change also shifts the oxygen dissociation curve to the left, leading to an increased affinity of the ferrous iron for oxygen and causing impaired oxygen release to tissues [1]. This process ultimately results in a state of functional anemia without a decrease in Hb concentration [2].

Methemoglobinemia can result from either congenital or acquired mechanisms. Congenital cases can be caused by variants of the CYB5R3 gene, or by heterozygosity for the pathogenic variant of the globin gene that generates an M hemoglobin [3,4]; however, the congenital forms rarely present with tissue hypoxia due to the presence of compensatory erythrocytosis. Acquired forms are the most common and can occur with drugs such as dapsone, antimalarials, topical benzocaine, nitrates, nitrites, rasburicase, and alanine dyes. Depending on the proportion of circulating MetHb, the condition can be severe or fatal. This diagnosis can be suspected when an adult or child presents with unexplained cyanosis or hypoxia that does not resolve with supplemental oxygen. MetHb can be detected on most arterial blood gas machines, but pulse oximetry and venous blood gas are not of use.

The treatment of methemoglobinemia should be initiated if a patient is symptomatic, with the first choice typically being methylene blue (MB) unless there are contraindications. Symptoms are typically seen once MetHb levels are greater than 10%. Observation is appropriate for patients who are asymptomatic with MetHb levels below 30%. MB is contraindicated in individuals who have glucose 6 phosphate dehydrogenase (G6PD) deficiency and in individuals who take serotonergic drugs. Alternatives to MB are exchange transfusion therapy, hyperbaric oxygenation, or high-dose ascorbic acid.

The authors present a case of a 39-year-old female who presented to the emergency department with hypoxia and cyanosis. She was found to have chocolate-colored blood, and arterial blood gas revealed a level of 33% MetHb. She was found to have been taking phenazopyridine for a urinary tract infection for the preceding two weeks. Phenazopyridine is a urinary analgesic medication available over-the-counter for the symptomatic relief of dysuria. It has been known to cause methemoglobinemia; however, there are few cases of this present in the current literature. MB was contraindicated in this case as the patient was taking citalopram for depression, and she refused an exchange transfusion due to religious beliefs as a Jehovah’s witness. Given a lack of access to hyperbaric oxygen, the patient was treated with high-dose ascorbic acid, with the resolution of her symptoms.

## 2. Case Report

A 39-year-old female presented to the emergency department by means of emergency medical transportation due to progressive shortness of breath for one week and bluish discoloration of the extremities. She did not have any significant past medical history apart from depression, but her family mentioned that she had been dealing with a urinary tract infection (UTI) for the preceding 2 weeks. The patient reported shortness of breath at rest, which worsened with activity, as well as headaches, nausea, vomiting, and dizziness. Her blood pressure was 118/67 mmHg, her respiratory rate was 23 breaths per minute, and her oxygen saturation was 83% on room air. She was placed on 10 L/min of oxygen through a nasal cannula, but her oxygen saturation remained between 82% and 86%. She was then placed on bilevel positive airway pressure (BiPAP,) with a fraction of inspired oxygen (FiO2) of 100% and an oxygen flow rate of 15 L/min, which improved her oxygen saturation to 89–90%. Chest radiography and computed tomography (CT) were carried out, which did not show any acute abnormalities. A physical examination revealed mild tachypnea with cyanosis of the lips and extremities, most notably on her toes. Chest auscultation revealed symmetrical air entry without wheezing or crackles present.

A complete metabolic panel and a complete blood count were both negative for any significant findings. Urinalysis was positive for nitrates, leukocyte esterase, white blood cells, and bacteria. A urine culture eventually resulted in being positive for Escherichia coli. COVID-19 and a respiratory viral panel were negative. Arterial blood gas displayed a pH of 7.52, pCO_2_ of 40 mmHg, pO_2_ of 381 mmHg, and methemoglobin of 33%. A review of the patient’s medical history made it apparent that she was taking citalopram for depression and had been taking phenazopyridine for a UTI for the past two weeks. Her family said she acquired the phenazopyridine over-the-counter, and that she did not go to her primary care physician (PCP) because she thought her symptoms were resolving. She had taken 2 99.5 mg tablets 3 times a day for 2 weeks. A review of the online literature revealed that phenazopyridine was a potential cause of methemoglobinemia. The patient could not be treated with MB due to the risk of inducing serotonin syndrome, as she was taking the serotonergic medication citalopram. She and her family refused an exchange transfusion due to religious beliefs as Jehovah’s witnesses and expressed that “she would rather die” than receive blood products. Subsequently, hematology was consulted, and the patient was placed in the intensive care unit (ICU) and started on 5 g of ascorbic acid every 12 h for 5 total doses. She was also started on 1 g of ciprofloxacin once daily for seven days.

**Figure 1:**
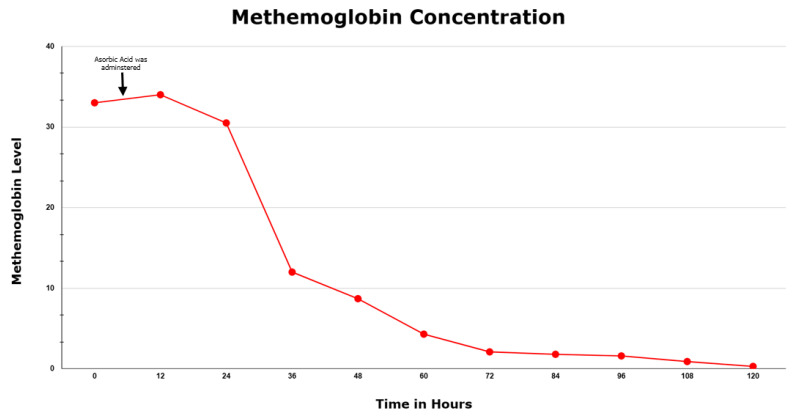
Methemoglobin concentration during the patient’s hospital course.

Initially, during the first 24 h the patient did not show any response, but she incrementally improved thereafter in both her MetHb level and her oxygen requirement (Figure 1). She was ultimately discharged home after 5 days in the ICU and advised to follow up with her PCP after her symptoms completely resolved. This paper was written according to CARE case report guidelines, and the patient’s permission was received prior to writing this report.

## 3. Discussion

Acquired methemoglobinemia is a medical emergency, and diagnostic clues such as known exposure to risk factors, cyanosis out of proportion to pulse oximetry, pulse oximetry of approximately 85 percent saturation of oxygen that does not improve with supplemental oxygen, and dark red to blue blood that does not turn red on oxygenation should be looked out for. The diagnosis is confirmed by measuring the level of methemoglobin in the blood. After exposure to an oxidizing substance that induces MetHb formation, the onset of symptoms is typically abrupt. The symptoms range from dyspnea, cyanosis, headache, fatigue, irritability, lethargy, shock, respiratory depression, seizures, or coma, which can be fatal if left untreated.

The severity of symptoms will correlate with the MetHb level; severe symptoms typically appear with a MetHb level above 30%, and there may be tachypnea, confusion, and loss of consciousness. Cyanosis may be evident with a methemogloblin level as low as 10% [5]. The characteristic chocolate-colored blood presents once the level reaches 15%. Once methemoglobinemia reaches 20%, the typical symptoms include anxiety, lightheadedness, and headache [2]. After levels reach beyond 50%, there is a risk of seizures, dysrhythmias, metabolic acidosis, and coma. Once levels are above 70%, the result is usually fatal [6].

Phenazopyridine, utilized as a urinary analgesic, is an azo dye recommended to be taken at a dose of 100 to 200 mg three times a day with a maximum duration of two days for symptom relief. Over-the-counter in the United States, the maximum dose obtainable is 199 mg per dose. It has been noted as the cause of methemoglobinemia in a few case reports [7,8,9,10,11,12,13]. In most cases in the current literature, patients were taking the drug either for an extended period of time beyond the recommended two days or at a larger dosages than the recommended daily dose. Acquired methemoglobinemia is seen in less than < 1% of patients who are treated with phenazopyridine, but the risk is increased in those who exceed the clinical recommendations [14].

MetHb levels are typically kept between 1 and 3% via cytochrome b5 reductase, NADPH methemoglobin reductase, or non-enzymatic processes [15]. Methemoglobinemia occurs in acquired cases, such as with the usage of phenazopyridine, when MetHb builds up beyond the capacity of intrinsic NADPH methemoglobin reductase [16]. Other medications associated with methemoglobinemia are local anesthetics, dapsone, and other drugs that increase oxidative stress. To reverse methemoglobinemia, ferric hemoglobin must be reduced to the ferrous state by one of two pathways. The pathway utilized in normal physiology is through cytochrome b5 reductase, which catalyzes an NADH-dependent reaction [17]. An alternative pathway is that through which methylene blue and ascorbic acid (extrinsic electron acceptors) act, and consists of NADPH generated by G6PD in the hexose monophosphate shunt (HMP) to reduce ferric hemoglobin to ferrous hemoglobin [18].

The primary treatment of methemoglobinemia is methylene blue, which is a serotonergic drug that is converted into leucomethylene blue. This then allows for the reduction of the heme group from methemoglobin to hemoglobin though the NADPH-dependent HMP shunt [19]. This reduction depends on NADPH, which is in turn generated by G6PD. In individuals who are deficient in G6PD, methylene blue is ineffective and can potentially precipitate hemolysis. MB is a potent monoamine oxidase inhibitor; therefore, individuals who are taking serotonergic medications should not receive MB due to the risk of potentiating serotonin syndrome.

An alternative treatment is ascorbic acid, which can be utilized when MB is unavailable or contraindicated. There have been a few cases in the current literature detailing the use of ascorbic acid in cases of methemoglobinemia; these are listed in Table 1 [20,21,22,23,24,25,26,27,28]. Up to 10 g can be administered in a single dose or in divided doses. The expected response can occur in one to three days, depending on the blood levels of MetHb [29]. In this case, the authors utilized ascorbic acid due to contraindications for the use of MB, but this can also be utilized in individuals who have known or suspected G6PD deficiency, as it acts independent of the NADPH pathway [19]. The mechanism is unknown but is thought to be mediated by ascorbic acid’s antioxidant properties [29]. In high doses, ascorbic acid may precipitate oxalate stones and even renal failure; therefore, the renal function of patients should be closely monitored. The authors do not recommend the usage of ascorbic acid in individuals who are able to receive MB, as MB has been extensively studied and proven to be effective in treating methemoglobinemia. Alternative treatments for use when ascorbic acid or MB are not appropriate include exchange transfusion therapy and hyperbaric oxygenation [30,31].

Acquired methemoglobinemia can be life-threatening if unrecognized and, therefore, must be identified and treated promptly. In hypoxic patients with a lack of improvement in oxygenation despite high-flow supplementation without an apparent cause, an elevated saturation gap of greater than 5% between arterial blood gas and pulse oximetry can help aid in the diagnosis of possible methemoglobinemia. Patients can be diagnosed with a positive blood MetHb level detected via an arterial blood gas analysis. A thorough review of medications taken by those diagnosed is a necessary step in identifying the disease etiology, as some medications have been shown to induce methemoglobinemia. Phenazopyridine is a commonly used over-the-counter medication, but awareness should be spread about the risk of methemoglobinemia in patients who take it against guidelines. A high index of suspicion of methemoglobinemia is needed in patients who present with hypoxia after prolonged usage of phenazopyridine. Our case is unique, as the patient was unable to receive MB—the standard treatment—since she was taking another serotonergic medication. The authors conclude that more research is needed regarding the utility of ascorbic acid in the treatment of methemoglobinemia, as no set guidelines currently exist for its dosage.

## Figures and Tables

**Table 1:** A Comparison of Cases Which Have Received Ascorbic Acid for The Treatment of Methemoglobinemia.

Author	Cause of Methemoglobinemia	Initial Methemoglobinemia Percentage	Ascorbic Acid Dosing and Treatment	Why Methylene Blue Was Not Utilized
Hamzaoui et al. [21]	Dapsone	13.70%	1 g of oral ascorbic acid every 12 h for 2 days and a loading dose of 50 g of activated charcoal orally, followed by 25 g every 6 h orally	It was not available
Kabir et al. [22]	Dapsone	17.70%	10 g of IV ascorbic acid every 6 h for 4 days, and was then switched to oral vitamin C	Unknown glucose-6-phosphate dehydrogenase status
Reeves et al. [23]	Rasburicase-induced	14.50%	5 g of IV ascorbic acid every 6 h for 3 days	Glucose-6-phosphate dehydrogenase deficiency
Sahu et al. [24]	Dapsone	18.30%	1 g of IV ascorbic acid every 12 h for 7 days	It was not available
De Crem et al. [25]	Primaquine	33.70%	1 g of IV ascorbic acid 4 times daily for 7 days	Patient was taking trazodone
Topal et al. [26]	Pilocarpine	24.50%	3 g of IV ascorbic acid over the course of 24 h	It was not available
Powell et al. [27]	Lava lamp poisoning	Over 30%	5 g of IV ascorbic acid over the course of 24 h	Patient was taking trazodone and duloxetine
Asif et al. [28]	Clofazimine	26.70%	0.5 g of oral ascorbic acid every 6 h (2000 mg/day) and 600 mg of oral N-acetylcysteine 600 mg every 8 h	It was not available
Kilicli et al. [29]	Prilocaine	14.10%	3 g of IV ascorbic acid over the course of 24 h	It was not available
Menakuru et al.	Phenazopyridine	33.0%	5 g of IV ascorbic acid every 12 h for 2.5 days	Patient was taking citalopram

## Data Availability

No additional data are available; all data are present in the report.
